# YAP1 regulates prostate cancer stem cell-like characteristics to promote castration resistant growth

**DOI:** 10.18632/oncotarget.23014

**Published:** 2017-12-07

**Authors:** Ning Jiang, Binghu Ke, Kim Hjort-Jensen, Diego Iglesias-Gato, Zhun Wang, Pengcheng Chang, Yang Zhao, Xiaodan Niu, Tao Wu, Bo Peng, Mingdong Jiang, Xiaoshi Li, Zhiqun Shang, Qiang Wang, Chawnshang Chang, Amilcar Flores-Morales, Yuanjie Niu

**Affiliations:** ^1^ Tianjin Institute of Urology, The Second Hospital of Tianjin Medical University, Tianjin, China; ^2^ Department of Drug Design and Pharmacology, Københavns Universitet, Faculty of Health and Medical Sciences, University of Copenhagen, Copenhagen, Denmark; ^3^ Danish Cancer Society, Copenhagen, Denmark; ^4^ University of Minnesota, Minneapolis, MN, USA; ^5^ University of Rochester, Rochester, NY, USA

**Keywords:** prostate cancer, YAP1, androgen receptor, DNA methylation, castration resistant prostate cancer

## Abstract

Castration resistant prostate cancer (CRPC) is a stage of relapse that arises after various forms of androgen ablation therapy (ADT) and causes significant morbidity and mortality. However, the mechanism underlying progression to CRPC remains poorly understood. Here, we report that YAP1, which is negatively regulated by AR, influences prostate cancer (PCa) cell self-renewal and CRPC development. Specifically, we found that AR directly regulates the methylation of YAP1 gene promoter via the formation of a complex with Polycomb group protein EZH2 and DNMT3a. In normal conditions, AR recruits EZH2 and DNMT3a to YAP1 promoter, thereby promoting DNA methylation and the repression of YAP1 gene transcription. Following ADT treatment or when AR activity is antagonized by Bicalutamide or Enzalutamide, YAP1 gene expression is switched on. In turn, YAP1 promotes SOX2 and Nanog expression and the de-differentiation of PCa cells to stem/progenitor-like cells (PCSC), which potentially contribute to disease recurrence. Finally, the knock down of YAP1 expression or the inhibition of YAP1 function by Verteporfin in TRAMP prostate cancer mice significantly suppresses tumor recurrence following castration. In conclusion, our data reveals that AR suppresses YAP1 gene expression through a novel epigenetic mechanism, which is critical for PCa cells self-renewal and the development of CRPC.

## INTRODUCTION

The development of lethal CRPC is driven by complex genetic and epigenetic mechanisms that remain poorly understood [1–5]. In a recent study, we identified the involvement of YAP1, a key effector of the Hippo signaling pathway in the regulation of CRPC growth [6]. YAP1 is a transcriptional co-activator that regulates the functions of the TEAD and SMAD families of transcription factors to promote the expression of genes involved in cell cycle, differentiation and metabolism [7, 8]. The canonical mammalian Hippo pathway consists of MST1/2 and LATS1/2 kinases, which cooperate with SAV1 and MOB1 to phosphorylate, thereby inhibit YAP1 function [9, 10]. We already provided the first evidence that YAP1 activity is under androgen control [6]. Androgen treatment represses YAP1 expression in PCa cells resulting in reduced cell proliferation and invasion capacity. Moreover, nuclear YAP1 accumulates in CRPC and the inhibition of YAP1 function with Verteporfin reduces the growth of androgen insensitive tumors *in vivo* [6]. More recently, it was shown that YAP1 could act as a coactivator of the AR in conditions of reduced hormonal levels [11]. Moreover, in mouse models of PCa, YAP1 can also regulate the recruitment of polymorphonuclear myeloid-derived suppressor cells, which promotes tumor growth [12]. Given these initial findings, it is clear that YAP1 mode of regulation and mechanism of action in urological malignancies merits additional studies.

Here, we explore the mechanisms behind androgen regulation of YAP1 function in prostate and provide multiple lines of evidence that demonstrate how AR directly represses YAP1 gene transcription through DNA methylation. In addition, we showed that YAP1 plays a critical role in regulating proliferation of prostate cancer progenitor-like cells to contribute to the growth of CRPCa.

## RESULTS

### Androgen-AR signaling suppresses YAP1 gene expression

**Figure 1:**
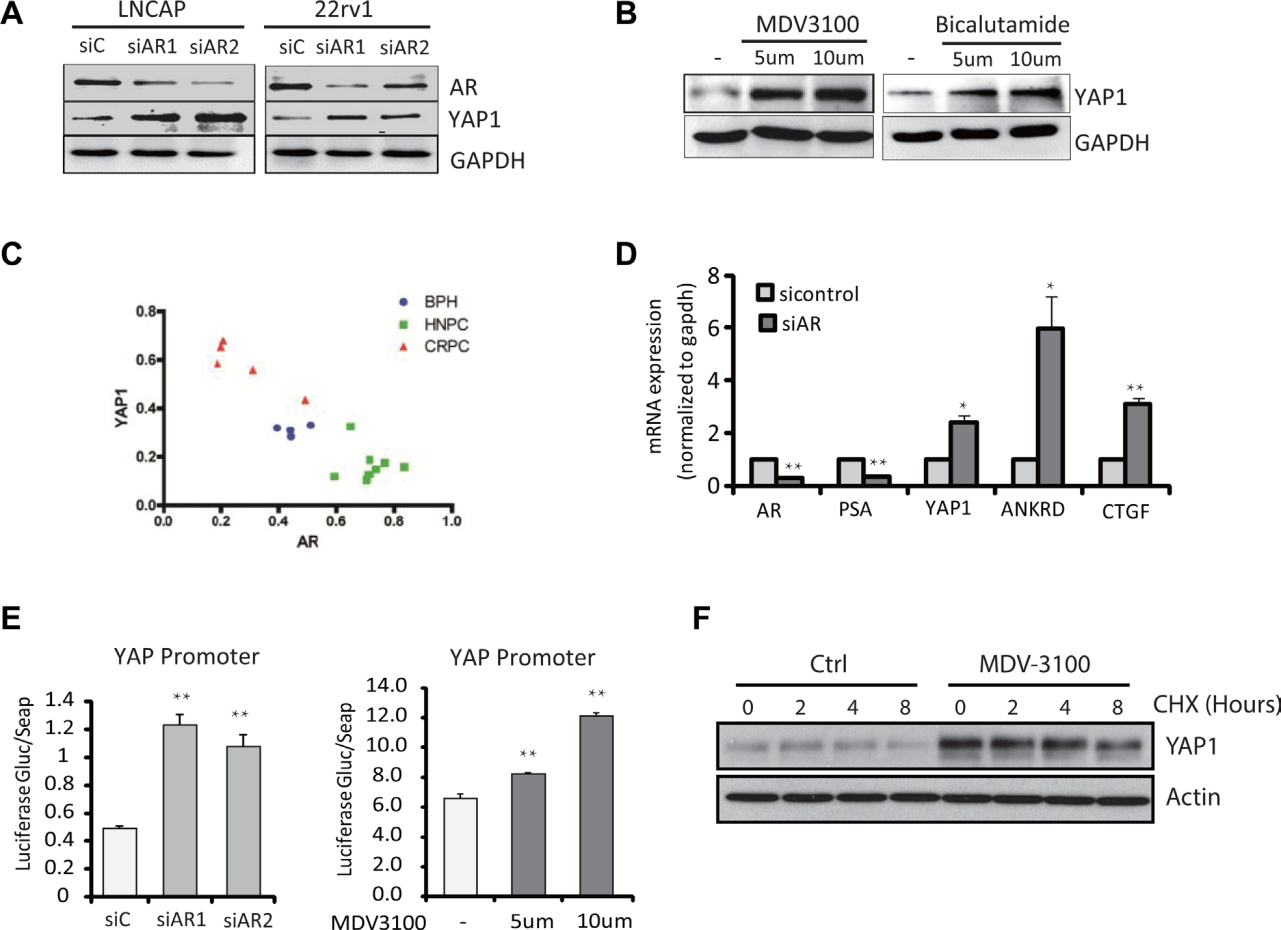
Androgen-AR signaling suppresses YAP1 gene expression (**A**) The levels of YAP1 protein were measured by Western Blot in LNCaP and 22RV1 cells upon AR knockdown. (**B**) The levels of YAP1 protein were measured in LNCaP cells treated for 24 h with the AR antagonist MDV3100 (enzalutamide, 100 nM) and Casodex (Bicalutamide, 10mM). (**C**) Quantitation of the immunofluorescence staining of AR (red) and YAP1(green) in BHP, HNPC and CRPC tissue samples from prostate cancer patients. (**D**) qPCR measurements of AR, PSA, YAP1, ANKRD and CTGF mRNA expression in siAR transfected LNCaP cells. Statistical analysis used Student’s T-Test, (^*^p < 0.05,^**^p < 0.01). Error bars represent the SD of triplicate measurements. (**E**) YAP1 promotor driven luciferase activity in LNCaP cell transfected with an siRNA targeting the AR or treated with AR antagonist MDV3100. The signal was quantified and statistical significance analyzed by Student’s T-Test, (^*^p < 0.05,^**^p < 0.01). (**F**) The levels of YAP1 proteins were measured by Western Blot in LNCaP after MDV3100 and CHX treatments for the indicated times.

In order to investigate the mechanisms responsible for the regulation of YAP1 expression by androgens in prostate, firstly, we investigated the regulation of YAP1 by AR *in vitro*. We found increased YAP1 protein expression levels following AR knockdown in PCa cells (Figure 1A) while treatment of LNCaP cells with AR antagonists MDV3100 and Bicalutamide also upregulated YAP1 protein levels significantly (Figure 1B) [6]. On the other hand, overexpression of ectopic AR in LNCaP cells led to reduced YAP1 expression levels (Supplementary Figure 1A). Finally, we observed that YAP1 levels in clinical samples are negatively correlated with AR levels (Figure 1C, Supplementary Figure 1B). Overall, these experiments demonstrate that AR regulates nuclear YAP1 protein levels in human and mouse prostate tissue.

To further investigate the mechanism of YAP1 repression by AR, we analyzed YAP1 mRNA content upon AR knockdown (Figure 1D). The mRNA levels of YAP1 and of down-stream target genes CTGF and ANKRD increased after AR inactivation, suggesting that AR may regulate YAP1 at the level of transcription. Indeed, the activity of a luciferase reporter driven by the YAP1 gene promoter increased upon AR knockdown or after treatment with AR antagonists (Figure 1E). On the other hand, androgen stimulation significantly inhibited the activity of a YAP1 promoter driven luciferase reporter (Supplementary Figure 1C). Inhibition of *de novo* protein synthesis by cyclohexamide treatment showed that the turnover of the YAP1 protein did not change significantly after androgen receptor inhibition (Figure 1F). Overall, these results strongly indicate that YAP1 regulation by androgen-AR signaling involves transcriptional repression.

### AR-mediated repression of YAP1 is associated with DNA methylation in the YAP1 promoter region

**Figure 2:**
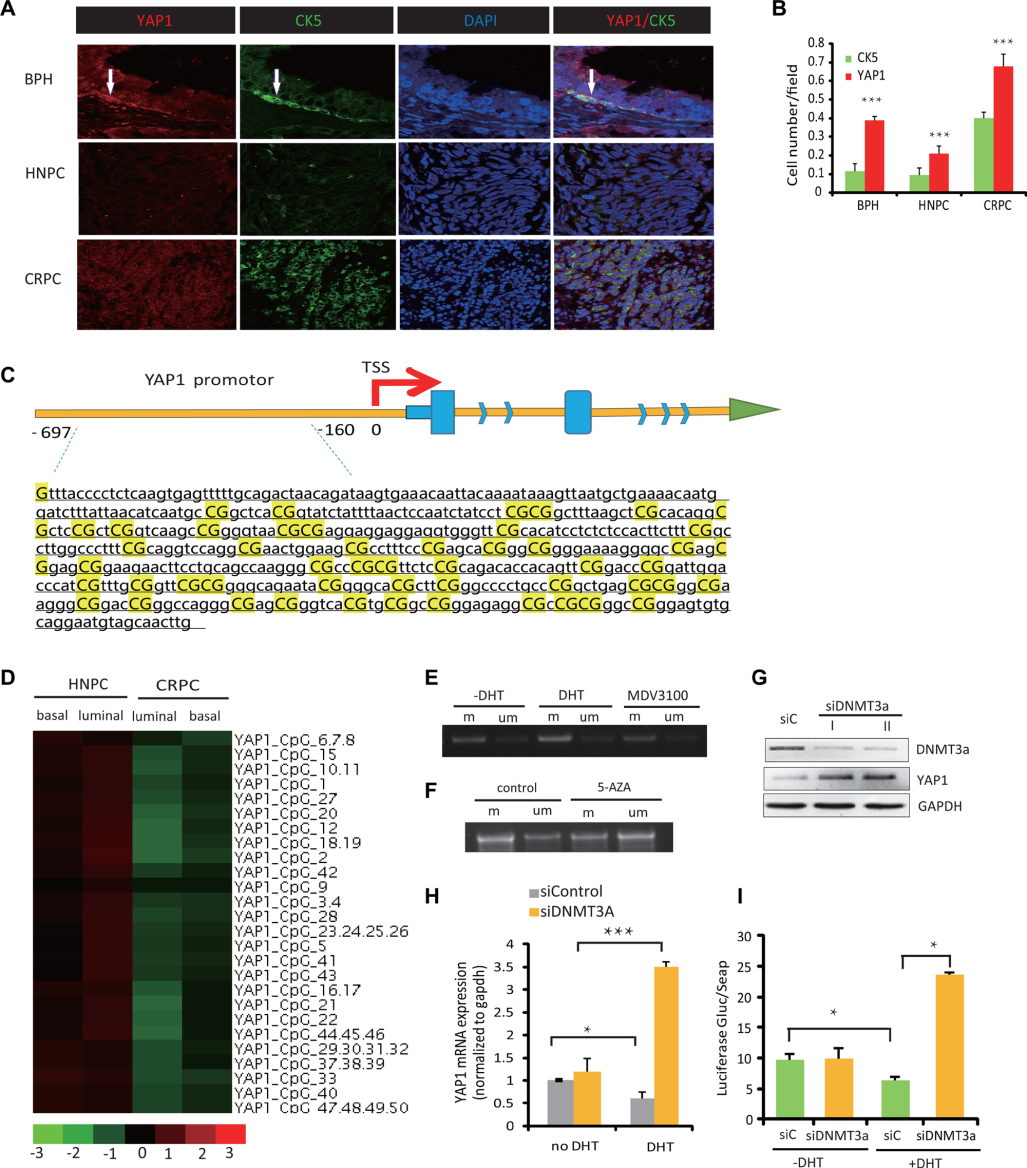
AR-mediated repression of YAP1 is associated with DNA methylation of the YAP1 promoter region (**A**) Immunofluorescence staining (IF) of YAP1 (red) and CK5 (green) in BHP, HNPC and CRPC tissue from human prostate cancer patients. Cell nuclei were visualized by DAPI staining. Representative stainings of YAP1 (red) and CK5 (green) in BHP, HNPC and CRPC tissue from human prostate cancer patients are shown. (**B**) Quantitation of YAP1 and CK5 expression from (A). (**C**) The schematic map of the YAP1 promoter-associated CpG island region. (**D**) Heatmaps showing the levels of YAP1 promoter methylation in four cell lines, HNPC cells (luminal and basal) and CRPC cells (luminal and basal). (**E**, **F**) DNA methylation PCR (MSP) show levels of YAP1 promotor methylation after treatment with DHT (10 nM), MDV3100 (100 nM) or 5AZA (5 μM). (**G**) Western blot analysis of YAP1 expression in LNCaP cell transfected with siRNA targeting DNMT3a (siDNMT3A). (**H**) qPCR measurement of YAP1 mRNA expression in siDNMT3a transfected LNCaP cells with or without DHT treatment. (**I**) YAP1 gene promoter driven luciferase reporter activity in LNCaP cell after treatment with DHT and upon transfection with siDNMT3a.

The normal prostate CK5^+^ basal type of epithelial cells express none or low levels of AR [13]. Therefore, we predicted that this cell population would be enriched for YAP1 nuclear expression and could serve to analyze the basis for YAP1 repression by the AR. Indeed, parallel analysis of YAP1 and CK5 protein expression in benign prostate hyperplasia (BPH), hormone naive PC (HNPC), and CRPC tissue revealed that in BPH, endogenous YAP1 is predominantly expressed in the nuclei of the AR-negative, CK5-positive basal epithelial cells (Figure 2A, 2B). YAP1 protein was also detected at lower levels in AR-expressing luminal cells, where the signal was diffused throughout of the cell. In line with our previous findings [6], immunofluorescence staining showed that YAP1 expression was robustly activated in CRPC compared with the BPH and HNPC tissues (Figure 2A, 2B). These findings were confirmed by Western blot analysis (Supplementary Figure 1D). Co-expression of YAP1 and CK5 was also found in CRPC tissue, again confirming the elevation of YAP1 expression in CK5-positive, AR-low basal-like type of PCa cells (Figure 2A, 2B).

It was previously reported that Yap1 gene transcription is regulated through epigenetic mechanisms in hepatocyte carcinomas [14]. Therefore, we hypothesized that the alterations of YAP1 expression in the different subtypes of epithelial cells (basal or luminal) are due to the variable methylation status of the YAP1 gene, promoter-associated CpG island regions (shown in Figure 2C). In order to test this hypothesis, we used cultured cells from primary xenografts obtained from fresh human prostate cancer tissue specimens. Through orthotopic inoculation of these cells into the prostate of intact or castrated nude mice, we established a model of prostate cancer, and confirmed via pathological H-E staining and immunohistochemistry that the tumors in nude mice were prostate cancer derived from human tissue. We cultured cells from tumors grown in either intact or castrated mice. Subsequently, we used flow cytometry to separated cell fractions from hormone treatment naïve (HNPC) and CRPC cells using CD49f and Trop2 antibodies and obtained luminal-like cells (CD49f-, Trop2+) and basal-like (CD49f+, Trop2+) cells from both HNPC and CRPC tumor cell cultures. The methylation profiles of the YAP1 promoter region in luminal and basal-like cells were investigated using methylation DNA sequencing. The difference of methylation status among various groups is significant and in favor of higher methylation levels in luminal cells than in basal cells (HNPC luminal cells (0.60 ± 0.032) > HNPC basal cells (0.47 ± 0.024), CRPC luminal cells (0.31 ± 0.024) > CRPC basal cells (0.20 ± 0.028) (*P* < 0.05, respectively)) (Figure 2D, Supplementary Table 1).

We also performed methylation-specific PCR (MSP) to amplify either methylated or unmethylated sulfite-modified DNA in the promoter-associated CpG islands of the YAP1 gene, (Supplementary Table 1). In LNCaP cells, MSP product increased after DHT treatment, indicating androgen signaling induces methylation at the YAP1 gene CpG islands (Figure 2E). Accordingly, when LNCaP cells were treated with MDV3100 the amount of MSP product decreased (Figure 2E). Treatment with 5-AZA (a DNA methylation inhibitor) diminishes the YAP1 CpG island methylation (Figure 2F). Because AR blockage is known to induce the expression of DNMT3a [16], we next studied how YAP1 expression is modulated by DNMT3a knockdown. YAP1 protein levels increased significantly upon DNMT3a downregulation (Figure 2G). We observed that siRNA mediated targeting of DNMT3a (Figure 2H) had little influence on YAP1 mRNA levels in the absence of androgens. In contrast, DHT treatment causes a significant increase in YAP1 expression in DNMT3a knockdown cells causing a clear reversal of androgen-driven repression of YAP1 gene expression and of YAP1 promoter driven luciferase activity (Figure 2H, 2I).

### The essential role of the AR/DNMT3a/EZH2 complex in YAP1 transcription

**Figure 3:**
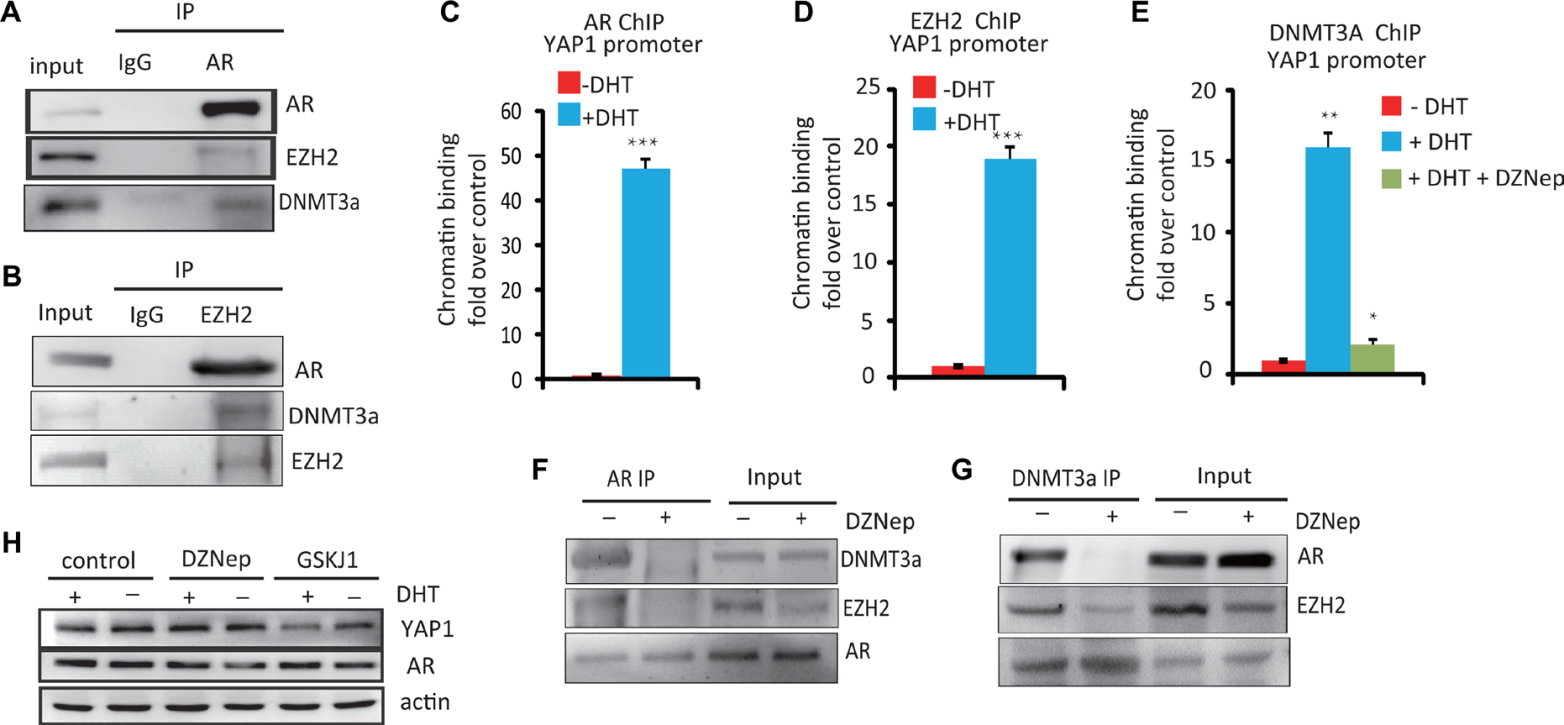
The essential role of the AR–DNMT3a and EZH2 complex in the regulation of YAP1 expression (**A**) Immunoprecipitation of AR in LNCaP cells followed by immunoblot analysis of EZH2, DNMT3a. IgG represents a control antibody used for IPs. (**B**) Co-IP of EZH2 and AR, DNMT3a in LNCaP cells (**C**) ChIP–PCR analysis of AR on YAP1 promoter. LNCaP cells were hormone starved for 12 h and then treated with ethanol, R1881 (10 nM) or DZNep (10 μM) for 6 h. Cells were then harvested for ChIP and analyzed by qPCR using. (**D**) ChIP–PCR analysis of EZH2 binding to the YAP1 gene promoter. (**E**) ChIP–PCR analysis of DNMT3a binding to the YAP1 gene promoter. LNCaP cells were treated with ethanol, R1881 or DZNep as indicated (**F**) Immunoprecipitation of AR in LNCaP cells treated with DZNep followed by immunoblot analysis of AR, EZH2 and DNMT3a. (**G**) IP with DNMT3a Ab in LNCaP cell treated with DZNep (10 μM) followed by immunoblot analysis of EZH2 AR and DNMT3a. (**H**) LNCaP cells were hormone starved for 12 h and then treated with ethanol or R1881 (10 nM), DZNep (10 μM) and GSKJ1(10 μM) for 8 h, WB analysis of YAP1 and AR protein expression.

Several studies have shown that the Polycomb repressive complex 2 (PRC2) subunit EZH2 and DNMT3a can cooperate for transcriptional repression [15]; while the interaction between AR and EZH2 has recently been shown to be important for regulating genes in prostate cancer development [16]. Therefore, we hypothesized that AR may recruit the EZH2, DNMT3a complex to repress YAP1 expression in PCa cells. To verify this hypothesis, we first investigated the interaction between AR and DNMT3a or EZH2 in LNCaP cells by co-immunoprecipitation (Co-IP) experiments. As show in Figure 3A, 3B and 3F, the AR forms a stable complex with DNMT3a and EZH2. Moreover, AR EZH2 and DNMT3a are recruited to the YAP1 gene promoter in response to androgen treatment (Figure 3C–3E) Interestingly, the ability of AR to form a complex with EZH2 and DNMT3a and the recruitment of DNMT3a to the YAP1 promoter was abolished by treatment with a EZH2 inhibitor, DZNep (Figure 3E–3G). DZNep treatment also lead to a significant increase in the expression of YAP1 in the presence of androgens (Figure 3H) Likewise, the H3K27 demethylase inhibitor GSK-J1 increases H3K27me3 levels (Supplementary Figure 1E) leading to decreased YAP1 expression both in the presence or absence of androgens (Figure 3H). Thus, our data shows that an active PRC2 complex is required for androgen-induced, DNMT3A-mediated repression of YAP1 expression. Together, the results indicate that a protein complex containing AR, EZH2, and DNMT3a mediates the androgen-driven epigenetic repression of the *YAP1* gene locus.

### YAP1 is required to sustain self-renewal and dedifferentiation of prostate cancer cells both *in Vitro* and *in Vivo*

**Figure 4:**
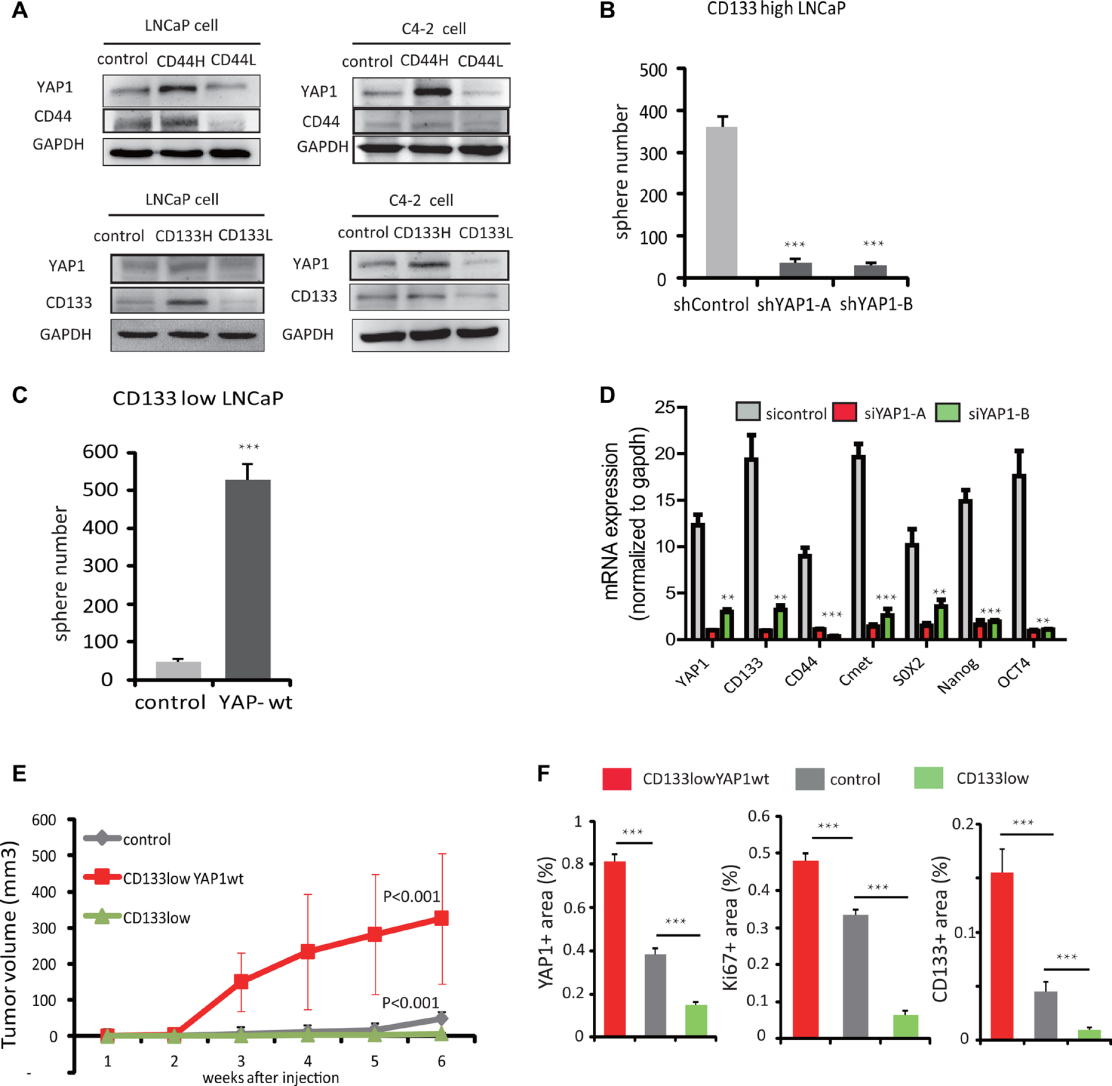
YAP1 is required to sustain self-renewal and dedifferentiation of prostate cancer cells *in vitro* **and** *in vivo*. (**A**) LNCaP cells and C4-2 cell subpopulations were isolated using magnetic sorting with antibodies recognizing stem cell markers, CD133 and CD44. Protein levels of YAP1, CD133 and CD44 were analyzed by Western Blot in control and CD133 or CD44 high or low cells. (**B**) Quantitation of sphere formation capacity of C4-2, CD133^high^ cells transfected with sh-control or sh-YAP1 expression vectors. (**C**) Quantitation of sphere formation capacity of C4-2, CD133^low^ cells transfected with either control or YAP1-wt expression vectors. (**D**) mRNA expression of stem cell marker genes: CD44, CD133, SOX2, OCT4, Nanog, C-met and YAP1 by qPCR after siRNA mediated knockdown of YAP1 expression in C4-2 cells. (**E**) CD133^low^ cell, CD133^low^-YAP1wt expressing cell and LNCaP cell were transplanted into nude mice subcutaneously and tumor growth was monitored daily. Growth curves are shown. *P* values were determined by paired t test. ^*^p < 0.05, ^**^p < 0.01, ^***^p < 0.001 (**F**) Quantitation of YAP1, Ki67 and CD133 protein expression in mice tumors using IHC. See also Supplementary Figure 2.

Adult PCSCs are thought to reside in the basal compartment because basal cells can survive androgen ablation and give rise to luminal cells [17, 18]. Given our previous findings that nuclear YAP1 expression is enriched in basal cells, we hypothesized that YAP1 may contribute to tumor growth through modulation of PCa progenitor-like cells. In order to test this hypothesis, we isolated stem/progenitor-like cells from LNCaP and C4-2 cell cultures using antibodies specific for the stem cell markers CD133 and CD44 [19] and investigated YAP1 expression in these cell subpopulations. We observed higher YAP1 expression in the CD133^high^ and CD44^high^ stem/progenitor-like cells as compared to unselected control cells and to CD133^low^ or CD44^low^ cells (Figure 4A).

To determine if YAP1 regulates the maintenance of PCa stem/progenitor cells, we tested whether YAP1 knockdown altered the ability of PCSCs to self-renew. We silenced the YAP1 gene using a YAP1 shRNA targeting vector and upon culturing multiple clones of YAP1 reduced C4-2 cells, we observed a decreased percentage of sphere forming cells. These prostate spheres were characterized by smaller, less dense morphologies compared with control cells (Supplementary Figure 1F). We also knocked down YAP1 from CD133^high^ LNCaP cells to check their ability to form spheres when cultured. We found that YAP1-reduced CD133^high^ LNCaP cells formed significantly fewer spheres than control cells (Figure 4B). On the other hand, we overexpressed YAP1 in CD133^low^ LNCaP cells and found an increased number of prostate spheres (Figure 4C). We also examined the expression of bona fide stem cell markers, including Oct4, Nanog, C-met and Sox2 using real-time PCR. Consistent with the previously described morphological changes, the mRNA expression levels of Oct4, Nanog, C-met and Sox2 strongly decreased in YAP1 knockdown cells compared to control PCSC (Figure 4D). Altogether, these data suggest that YAP1 is required to sustain self-renewal.

As CD133^low^ LNCaP cells express low levels of YAP1, we stably transfected these cells with a YAP1 cDNA expression vector and generated YAP1 overexpressing CD133^low^ LNCaP cells, named CD133^low^ YAP1wt. Then, we explored the *in vivo* tumorigenicity of parental LNCaP, CD133^low^ and CD133^low^ YAP1wt LNCAP cells. Although parental and CD133^low^ LNCP cells exhibited reduced capacity to generate tumors, YAP1 transfected CD133^low^ LNCAP cells clearly showed increased tumor forming capacity (Figure 4E). Next, we analyzed the expression of YAP1, of stem cell markers CD133, Nanog and SOX2 and of proliferation marker Ki67 in the xenograft tumors originated from these three cell lines using IHC staining (Figure 4F and Supplementary Figure 2). We found the expression of YAP1 to correlate with that of Nanog, SOX2 and Ki67, being higher in tumors of CD133^low^ YAP1wt than in tumors of LNCaP control cells and CD133^low^ cells (Figure 4F).

### YAP1-mediated stem cell renewal is dependent upon the TEAD-interaction domain of YAP1

**Figure 5:**
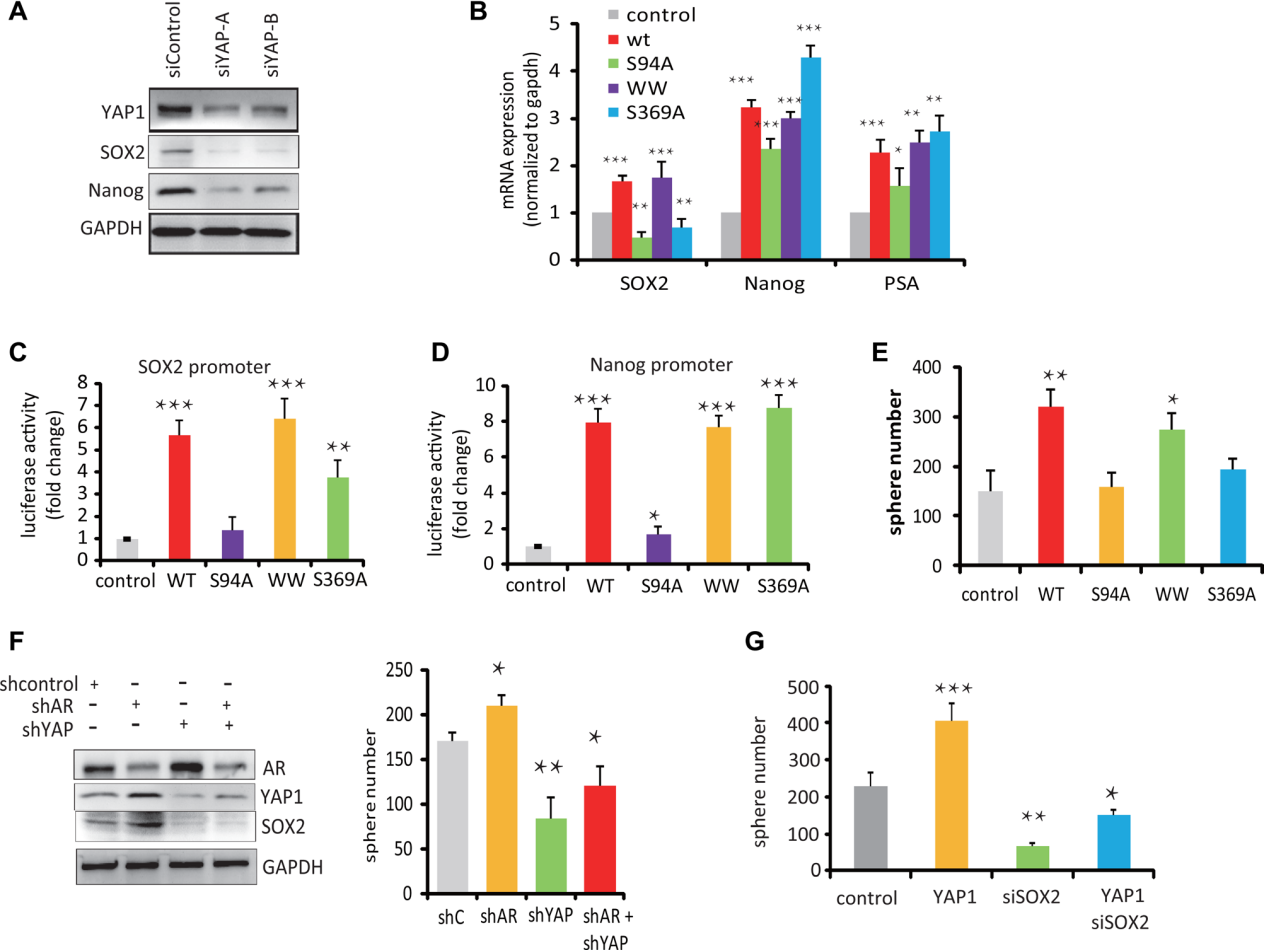
YAP1-mediated stem cell renewal depends on the TEAD-Interaction Domain of YAP1 (**A**) Western blot analysis of SOX2 and Nanog protein expression in C4-2 cells transfected with siYAP1 and siControl. (**B**) qPCR measurements of mRNA expression levels of SOX2, Nanog and PSA in C4-2 cells transfected with control, YAP1wt or YAP1-S94A, YAP1-WW and YAP1-S369A mutant expression vectors as indicated. (**C**, **D**) SOX2 and Nanog gene promoter driven luciferase reporter activity in C4-2 cell transfected with expression vector for YPA1-WT or YAP1 mutants as indicated. (**E**) Sphere formation activity of C4-2 cells transfected with expression vectors for YAP1-WT and YAP1 variants as indicated. (**F**) Sphere formation activity and Western blot analysis of AR, YAP1 and SOX2 expression in C4-2 cells transfected with shAR and shYAP vectors. (**G**) Sphere formation activity of C4-2 cells transfected with YAP1wt vector and siRNA targeting SOX2.

To further analyze the function of YAP1, we reduced the expression levels of YAP1 in C4-2 cells using YAP1 targeting siRNAs. In these cells, we found decreased SOX2 and Nanog protein expression (Figure 5A). We also investigated how YAP1 regulates the expression of these genes. Generally, YAP1 is composed of a TEAD-binding domain, a SH3-binding motif and two WW domains [20] (Supplementary Figure 3A). To identify which domain is responsible for promoting stem cell renewal, we expressed YAP1 domain-specific mutants in C4-2 cells to assess their effect on YAP1 function using previously characterized YAP1 TEAD-binding domain (YAP1-S94A), WW domain and SH3 domain (YAP1-S369A) mutants [21–23]. We observed a lower level of SOX2 and Nanog mRNA expression in YAP1-S94A expressing cells compared with cells transfected with YAP1-WT and other YAP1 mutants (Figure 5B). We also observed that YAP1-WT cells expressed increased levels of SOX2 and Nanog proteins compared to control cells, but cells expressing the YAP1-S94A mutant showed barely detectable levels of SOX2 and Nanog (Supplementary Figure 3B). To analyze whether YAP1 directly trans-activates SOX2 and Nanog gene promoters, we utilized a reporter assays to show that both SOX2 and Nanog gene promoters were strongly activated by transfection of wt-YAP1 and WW-YAP1 and S369A-YAP1 mutants, but to a much smaller extent by the YAP1-S94A variant (Figure 5C, 5D). These results show that the TEAD-binding domain of YAP1 plays a crucial role for the activation of SOX2 and Nanog gene transcription.

On a functional level, the number of spheroids formed by C4-2 cells over expressing wt-YAP1, and the YAP1-S369A mutants or YAP1-WW domain mutants significantly increased compared to control cells, suggesting that the WW domains and SH3 domain are not essential for YAP-mediated stem cell renewal in these conditions. On the other hand, cells expressing the YAP1-S94A mutant form significantly fewer spheres than control cells (Figure 5E). Together, these results suggest that integrity of the YAP1 TEAD-binding domain is require for YAP1 functions in prostate cancer cells renewal.

Interestingly, the capacity of LNCap cells to form spheres increased slightly upon transfection of an shRNA against AR, while shRNA against YAP1 decreased the number of spheres alone or in the background of AR knock-down (Figure 5F). Furthermore, knockdown SOX2 in C4-2 cells decreased the number of sphere forming cells even upon transfection with the YAP1-wt expression vector (Figure 5G). This suggests that the growth of PCSC after ADT is modulated largely through the actions of YAP1 on SOX2.

### Blocking YAP1 signaling increases the effectiveness of ADT

**Figure 6:**
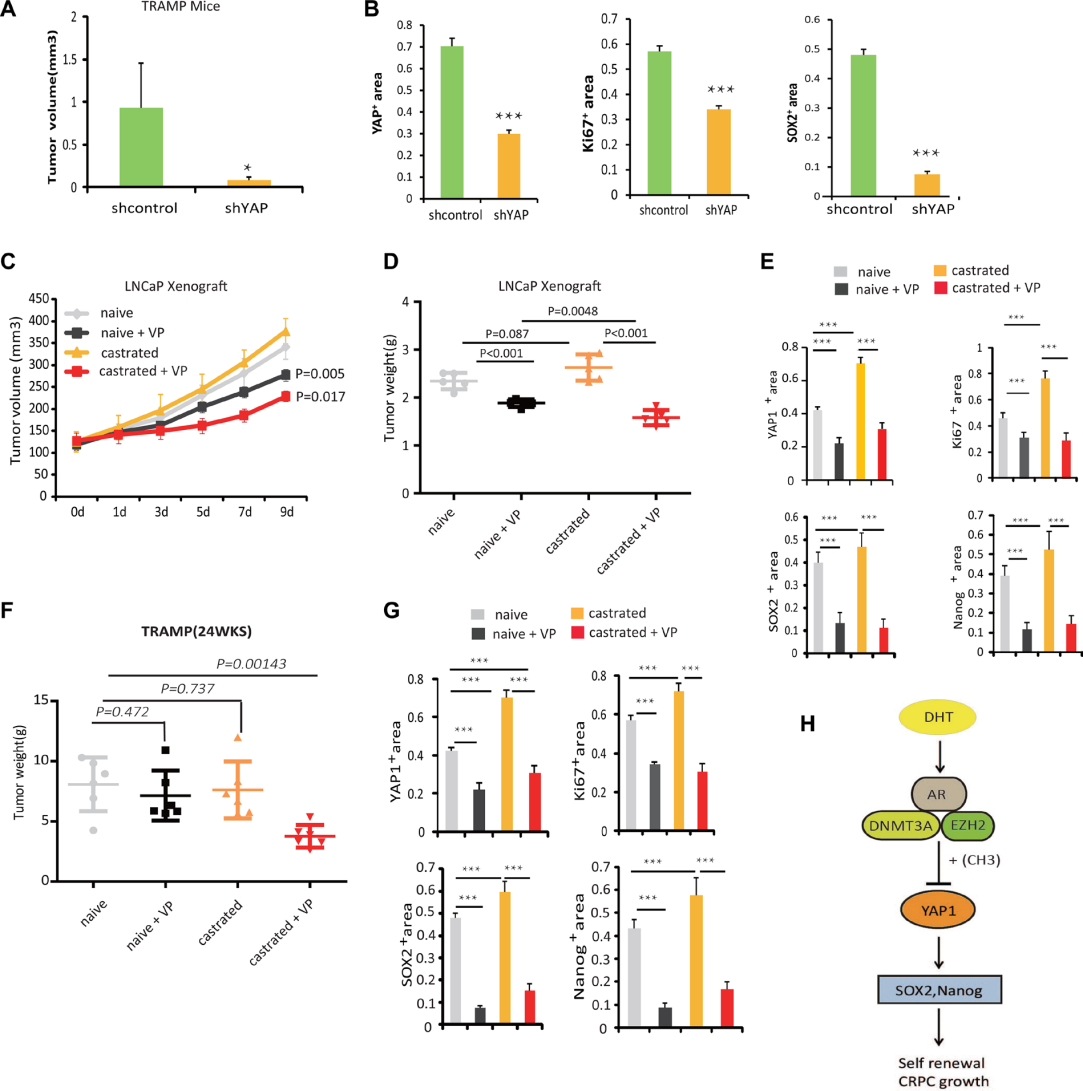
Blocking YAP1 signaling increases the effectiveness of ADT (**A**) 20 weeks old TRAMP mice were treated with lentivirus targeting YAP1 or with corresponding controls. The prostate tumors were harvested at 24 weeks of age. 3 mice were used in each group. (**B**) Quantitation of Ki-67, YAP1 and SOX2 expressions in TRAMP mice tumors from each group, specimens were obtained at 14 days post treatment. The IHC was scored according to number of cells expressing the indicated proteins and statistical analysis was performed using the non-parametric Kruskal-Wallis test in order to determine significance. See also Supplementary Figure 5. (**C**) Castration resistant and castration naïve LNCaP cells derived xenografts were injected subcutaneously in BalbC/ nude mice [8]. Tumors were allowed to grow until they reached a size of approximately 120 mm^3^. Subsequently, xenografted mice were randomized to receive either vehicle or 100 mg/kg Verteporfin (VP) daily for 9 days by intraperitoneal injections. The proliferation of tumors was monitored. 6 mice were used in each group. Statistical significance differences in tumor size were analyzed by Student’s T-Test, (^*^p < 0.05, ^**^p < 0.01). (**D**) Tumor weight in each group is shown. (**E**) Quantitation of Ki-67, YAP1, Nanog and SOX2 expressions in LNCaP xenograft tumors from each group. Specimens were obtained 10 days post-treatment. The IHC was scored according to the number of cells expressing the indicated proteins and statistical analysis was performed (non-parametric Kruskal-Wallis test) in order to determine significance. See also Supplementary Figure 5. (**F**) 12wks old TRAMP mice were randomized, castrated and then divided into naive and castration groups. At 18 weeks of age mice received 100 mg/kg Verteporfin (VP) daily, for 14 days by intraperitoneal injection. The prostate tumors were harvested at 24wks (*n* = 6 mice per group). (**G**) Quantitation of Ki-67, YAP1, Nanog and SOX2 expressions in TRAMP tumors from each group at 24wks. The IHC was scored according to number of cells expressing the indicated proteins and statistical analysis was performed (non-parametric Kruskal-Wallis test) in order to determine significance. ^*^p < 0.05, ^**^p < 0.01. See also Supplementary Figure 6, (**H**) Hypothetical model of AR- DNMT3a complex role in inhibition of YAP1 signaling. In prostate cancer cells, AR binds EZH2 and DNMT3a proteins to form a complex associated with the YAP1 promoter where DNMT3a induces DNA methylation to inhibit YAP1 gene transcription.

To further define whether increased YAP1 expression regulate CRPC development, lentivirus-shRNA mediated knockdown of YAP1 or treatment with YAP1 antagonist, Verteporfin [24] was used on LNCaP xenografts and TRAMP mice models. First, we used lentivirus shRNA-YAP1 to knockdown YAP1 expression in the prostate of TRAMP mice (20weeks) and analyzed the tumors after two weeks of treatment. We observed that tumors of the sh-YAP1 lentivirus group were smaller than control group (Figure 6A). IHC analysis confirmed that these tumors express less YAP1 and also show greatly reduced SOX2 and Ki67 protein levels, suggesting that both tumor cell proliferation and the number of progenitor/stem like cells are regulated by YAP1 expression *in vivo*(Figure 6B, Supplementary Figure 4).

Next, we tested the effects of Verteporfin, an FDA approved drug with known effects on the regulation of YAP1 content and activity [6]. LNCaP xenograft-bearing mice started to receive treatment until tumors reached a size of approximately 120 mm^3^, and TRAMP mice received YAP1- blocking treatment at week 22. The mice were castrated at week 12, the time they start to bear tumors [25]. When mice bearing LNCaP hormone sensitive tumors were treated with Verteporfin alone, the growth of these tumors showed a slight reduction in size (Figure 6C–6E, Supplementary Figure 5). Likewise, Verteporfin treatment of LNCaP-castration resistant tumors significantly suppressed tumor growth in comparison to both non-castrated control group and the castrated group (Figure 6C–6E, Supplementary Figure 5). We observed that Verteporfin treated tumors expressed reduced levels of YAP1, which are accompanied by diminished cell proliferation, and decreased expression of Ki67, SOX2 and Nanog markers. In the TRAMP model, we did not detect significant differences in tumor growth after castration or after Verteporfin treatments alone. In contrast, combined castration and Verteporfin treatments caused a significant reduction in tumor weight (Figure 6F, 6G, Supplementary Figure 6). Together, these results highlight the potential of targeting the YAP1 signaling to sensitize to anti-androgen therapy.

## DISCUSSION

In this study, we unveil key elements of the crosstalk between the AR and the Hippo signaling pathways and its implications for prostate carcinogenesis. Our study demonstrates that the AR assembles at the YAP1 promoter a repressive complex containing DNMT3A and EZH2 to suppress YAP1 transcription. Consequently, androgen deprivation therapy can induce YAP1 expression, which in turn regulates downstream factors such as SOX2 and Nanog to promote PCa stem/progenitor like cells and contribute to castration resistant prostate cancer growth (Figure 6H).

When activated by ligands, the AR binds to genomic androgen-response elements (AREs), which serve as a platform for recruitment of basal transcriptional factors and co-regulators to modulate gene transcription [26]. A few reports have studied AR inhibition of transcription. Some studies have hinted a indirect mechanisms of AR regulated transcriptional repression [27, 28], while others have shown the existence of direct mechanisms involving the EZH2 containing PRC2 repressive complex [29]. Using YAP1 as a model, we found that AR also inhibits gene expression by hormone induced recruitment of DNMT3a to the AR/EZH2 complex leading to methylation of promoter CpG islands resulting in epigenetic silencing. Consequently, ADT restores YAP1 expression due to the loss of AR-EZH2-DNMT3a association to the YAP1 promoter.

Our current understanding of the pathways the regulation of YAP1 proteins levels emphasizes the phosphorylation driven mechanisms that promote ubquitination dependent YAP1 degradation [9]. In contrast, androgen regulation of YAP1 levels seems to be mainly transcriptionally controlled because the rate of YAP1 protein degradation is not affected by AR blockage. The transcriptional control of YAP1 expression was also highlighted in a recent study by Nguyen LT. et al that show that probasin-driven ERG expression in mice leads to tumor formation in aged mice that are characterized by YAP1 overexpression [30]. In this model, the levels of active MST and LATS kinases do not change in comparison to control mice and paradoxically the levels of YAP1 phosphorylated in S127, which should lead to inhibition of YAP1 function also increase, suggesting that LATS1/2, MST1/2 actions on YAP1 may not be the primary factor controlling its activity *in vivo* [31]. Instead, the authors demonstrate that ERG can induce the transcriptional activation of the YAP1 gene by promoting H3 acetylation. Given that ERG is able to antagonize AR function in prostate cancer cells [30], it will be interesting to explore whether ERG regulation of YAP1 also relies on antagonizing the repressing actions of the AR/DNMT3a complex.

Prostate epithelium is composed of three types of cells: luminal, basal and neuroendocrine cells [32]. Several independent studies have verified that basal cells in human and murine prostates can generate all three prostate epithelial cell lineages [33], although prostate stem cell-like populations have been identified in both the basal and luminal layers [19, 34]. The Hippo signaling pathway plays a functional role on cancer cell differentiation in various organs [35]. In this study, we proved that YAP1 protein expression is elevated in CD133^high^ progenitor type of prostate cell and found that YAP1 overexpression inhibits PCSC differentiation and maintains PCSC features. Conversely, YAP1 knockdown results in loss of PCSC properties. Furthermore, YAP1 overexpression promotes CD133^low^ cells to de-differentiate into CD133^high^ stem cells-like cells that efficiently generate tumors when injected in mice. We also proved that YAP1 activation positively regulates the expression of Nanog and Sox2, while inhibition of YAP1 with Verteporfin or its knockdown by means of shRNA reduce cell proliferation alongside with diminished Sox2 expression. Therefore, it is possible that activated YAP1 in basal or intermediate cells enhances stem/progenitor cell expansion through transcriptional induction of Nanog and Sox2 [36].

Several non-overlapping mechanisms have been proposed to explain YAP1 effects on tumorgenesis, including promotion of epithelial-mesenchimal transition (EMT), resistance to anoikis, and induction of stem cell properties [10]. Here, we show that YAP1 contributes to the growth of castration resistance tumors via a de-differentiation mechanism leading to increased cell proliferation. Our findings does not exclude that additional YAP1 driven mechanisms such as the activation of AR [11] or immunomodulation [12] may also play a role in stimulating the growth of castration resistant tumors. Clearly, the exploration of YAP1 function is prostate cancer is in its infancy and additional studies are warranted. Given the results obtained in our pre-clinical models, it may be clinically relevant to explore novel therapeutic strategies to target AR and YAP1 pathways simultaneously in PCa treatment.

## MATERIALS AND METHODS

### Cell culture

The PCa cell line LNCaP-FGC was obtained from the American Tissue Culture Collection (Manassas, VA) and the C4-2 cells were obtained from the MD Anderson Cancer Center, University of Texas. Cells were maintained in RPMI 1640 medium supplemented with 10% FBS, 1% penicillin/streptomycin, and 1% glutamine. DHT was obtained from Amersham (Braunschweig, Germany), DZNep was from SELLECK (S71201) and MDV3100 was from Haoyuan Chemexpress (Shanghai, China).

### Plasmids

The hYAP1-Wt expression vector consists of the full-length YAP1 open reading frame cloned into pcDNA3.1 and was donated by Dr. Ka-Fai from Hong Kong SAR, PR China. YAP1 mutants were obtained from Addgene: YAP1-S94A (#33102), YAP1wt (#19045), WW mutant (#19048) and S369A (#18995). The Nanog-LUC reporter gene was donated by Dr. Yanhong Shi from CA, USA. The YAP1 promoter-LUC construct was bought from GeneCopoeia.

### Sphere formation assay

Single cell suspensions (1 × 10^3^, in 60 μl of medium) were mixed with 60 μl of cold Matrigel, and the mixture was placed along the rim of the 24-well plates. The culture plates were placed in a 37°C incubator for 10 min to let the mixture solidify, and 500 μl of medium was then added into the well. Sphere numbers were counted after 7–14 days under an Olympus light microscope, and size differences were also examined. A minimum of triplicate experiments were performed.

### Co-immunoprecipitation and western blotting

LNCaP cells were hormone starved for 12h and then treated with ethanol, R1881 (10nM) or DZNep (10mM) for 6 h. Cells were lysed in a buffer containing 150 mM KCl,75 mM Hepes, pH 7.5, 1.5 mM EGTA, 1.5 mM MgCl2, 10% glycerol, and 0.075% NP-40 supplemented with a cocktail of protease inhibitors [Roche, USA]. Extracts were pre-cleared using a mixture of protein A–Sepharose (CL-4B; GE Healthcare) or protein G-Sepharose (rec-Protein G-Sepharose 4B; Invitrogen) and immunoprecipitations were performed for 1 hour at 4°C. Immunoprecipitates were washed with lysis buffer, resuspended in sample buffer, boiled and analyzed by SDS-PAGE. Individual samples (40 μg of protein) were separated on 8–10% SDS polyacrylamide gel and transferred to PVDF membranes (Millipore, Billerica, MA). Membranes were blocked in a PBS-Tween 20 solution with 5% fat-free milk for 1h at room temperature, and subsequently the membranes were incubated with appropriate dilutions of specific primary antibodies, overnight at 4°C. After washing, the blots were incubated with HRP conjugated anti-rabbit or anti-mouse IgG for 1h. The blots were developed by enhanced chemiluminiscence (Vector Laboratories, Burlingame, CA) and visualized by Imager.

### RNA extraction and gene array analysis

RNA was extracted using TRIzol reagent (Invitrogen) according to the manufacturer’s instructions. RNAs (1 ug) were subjected to reverse transcription using Superscript III transcriptase (Invitrogen). The obtained cDNAs were used for qPCR using a Bio-Rad CFX96 system with SYBR Green. The primers used are listed in Table 1. Expression levels were normalized to the expression of the GAPDH gene.

**Table 1:** The expression levels of methylation in four cell lines, HNPC cell (luminal and basal), CRPC cell (luminal and basal)

name:YAP1		HNPC · basel	HNPC · luminal	CRPC · basel	CRPC · luminal
SampleID	CPG · Position	Y1	Y2	Y3	Y4
YAP1_CpG_1	102	0.47	0.55	0.22	0.3
YAP1_CpG_2	110	0.46	0.65	0.16	0.23
YAP1_CpG_3.4	139:141	0.45	0.57	0.25	0.27
YAP1_CpG_5	153	0.42	0.62	0.26	0.33
YAP1_CpG_6.7.8	161:166:170	0.47	0.41	0.27	0.21
YAP1_CpG_9	179	0.42	0.49	0.32	0.34
YAP1_CpG_10.11	187:189	0.47	0.56	0.19	0.28
YAP1_CpG_12	209	0.48	0.65	0.18	0.3
YAP1_CpG_15	260	0.51	0.55	0.21	0.29
YAP1_CpG_16.17	271:280	0.49	0.52	0.19	0.33
YAP1_CpG_18.19	286:290	0.47	0.62	0.17	0.27
YAP1_CpG_20	304	0.48	0.58	0.18	0.26
YAP1_CpG_21	308	0.46	0.56	0.16	0.32
YAP1_CpG_22	313	0.51	0.62	0.19	0.35
YAP1_CpG_23.24.25.26	338:342:344:351	0.45	0.61	0.25	0.29
YAP1_CpG_27	367	0.53	0.64	0.23	0.32
YAP1_CpG_28	373	0.49	0.63	0.24	0.28
YAP1_CpG_29.30.31.32	387:393:398:340	0.5	0.48	0.2	0.29
YAP1_CpG_33	412	0.58	0.55	0.22	0.27
YAP1_CpG_37.38.39	444:446:450	0.59	0.55	0.23	0.36
YAP1_CpG_40	457	0.59	0.55	0.24	0.32
YAP1_CpG_41	462	0.45	0.63	0.25	0.31
YAP1_CpG_42	472	0.48	0.6	0.26	0.33
YAP1_CpG_43	476	0.46	0.65	0.27	0.34
YAP1_CpG_44.45.46	483:487:491	0.48	0.64	0.18	0.35
YAP1_CpG_47.48.49.50	500:503:505:510	0.52	0.49	0.22	0.28

We design primer from -697 to -160 of YAP1 promtor enriched CG, and use a MassARRAY Compact MALDI-TOF(Sequenom), and spectra’s methylation ratioswe regenerated by the Epi-TYPER software v1.0 (Sequenom) in in four cell lines, HNPC cell (luminal and basal), CRPC cell (luminal and basal). Heatmaps showing higher methylation levels in luminal cells than those in basal cells.

### Luciferase assays

For the dual luciferase assay, LNCaP and C4-2 cells were plated into 12-well plates and co-transfected with 1 μg of the seap reporter gene construct and 15 pmol of YAP1 luciferase together with siRNA of other plasmids by using transfection reagent (Roche). Transfected cells were cultured and 24 h later the supernatants were collected for luciferase assay using Dual Luminescence assay kit (GeneCopoeia MD) according to the manufacturer’s instructions. To measure SOX2 and Nanog promoter driven luciferase activity, the Glo Luciferase Assay System (Promega) was used.

### Chromatin immunoprecipitation

LNCaP cells were grown in RPMI 1640 media (Invitrogen) supplemented with 10% charcoal-stripped FBS (CSF, HyClone, USA) for 12 h before stimulation with 1 × 10^−9^ M synthetic androgen R1881 or an equal volume of ethanol for 6 h. DNA cross-linking was performed by adding 1% formaldehyde into the cell cultures at room temperature for 10 min when glycine was added (0.125 M final concentration) for 5 min to stop the cross-linking reaction. Cells were lysed with a lysis buffer containing protease inhibitors and sonicated to shear genomic DNA to lengths between 200 and 1000 bp. One-tenth of the cell lysate was used for input control, and the rest was used for immunoprecipitation using AR, DNMT3a or EZH2 antibody. After collecting the immunoprecipitates using protein G-agarose columns, protein-DNA complexes were eluted and heated at 65°C to reverse the cross-linking. Following digestion with proteinase K, DNA fragments were purified using spin columns and analyzed using PCR for 35 cycles in a sequence of 94°C for 30s, 57°C for 30 s, and 72°C for 1 min. Specific primer sets were designed to amplify a target sequence within the human YAP1 promoter (Supplementary Table 2). PCR products were electrophoresed in a 1% agarose gel with ethidium bromide and visualized under ultraviolet light.

### Confocal fluorescence analysis

Cells were grown on Labtek II-CC2 Chamber slides (Nunc), fixed with 4% paraformaldehyde for 5 minutes, washed with PBS and blocking buffer (PBS with 5% BSA and 0.1% Triton X- 100), and then incubated overnight at 4°C in primary antibodies against YAP1, AR and CK5. Secondary antibodies were Donkey anti-mouse, -rabbit or -goat coupled to Alexa-350, -488 or -647 (Invitrogen). Cell nuclei were visualized with DAPI (Sigma).

### Animal studies

Four-week-old male Balb/c mice (HFK Bio-Technology Co. Ltd, Beijing) were injected subcutaneously with 2 × 10^6^ LNCaP cells suspended in 0.1 mL of Matrigel (BD Biosciences), and were implanted subcutaneously into the dorsal flank on both sides of the mice. Once the tumors reached an indicated size, the animals were randomized and received castration and/or daily treatment with 100 mg/kg body weight of Verteporfin by intraperitoneal injection for 9 days. Tumor volume was recorded by digital caliper and estimated using the formula LW^2^/2, where L = length of tumor and W = width. At the end of the study, mice were killed and tumors extracted and weighed. For TRAMP mice, we verified the genotypes by PCR using tail snip DNA as templates [30].Verified mice were randomized and received castration at 12wk-old age. For treatment, te lentiviral vector-expressing short hairpin (shRNA) YAP1 (1 × 10^8)^ was injected into the tumor Verteporfin, 100 mg/kg body weight was given daily by intraperitoneal injection for 14 days. At the end of the studies, mice were killed and tumors extracted and weighed. All procedures involving mice were approved by the University Committee on Use and Care of Animals at the Tianjin Medical University and conform to all international regulatory standards.

### Statistical analysis

Data is expressed as mean ± SD. Differences between samples were analyzed by Student’s *T*-test. *P*-values of 0.05 or less were considered significant.

## SUPPLEMENTARY MATERIALS FIGURES AND TABLES


