# Supplementary material for: YAP1 regulates prostate cancer stem cell-like characteristics to promote castration resistant growth
Source: Oncotarget. 2017 Dec 7;8(70):115054–67. doi: 10.18632/oncotarget.23014 (PMC5777753; doi:10.18632/oncotarget.23014)
Supplement: Supplementary file 1 [file oncotarget-08-115054-s001.pdf]

YAP1 regulates prostate cancer stem cell-like characteristics to promote castration resistant growth

SUPPLEMENTARY MATERIALS

Clinical samples

A total of 5 BPH, 10 androgen dependent PCa (ADPC) and 5 castration-resistant PCa (CRPC) specimens were obtained from patients who underwent radical prostatectomy or transurethral resection of the prostate at the Tianjin Medical University Hospital (Tianjin, China) and inspected by a certified pathologist for Gleason grading. The study was approved by the Ethics Committee of the Tianjin Medical University, and the Helsinki Declaration of Human Rights was strictly observed.

Primary antibodies and reagents

YAP1 (Cell Signaling Technology(CST), #12395 and #14074), AR (CST, #3202 and Abcam, ab74272), DNMT3a (CST,#3598), EZH2(Abcam,ab3748), H3K27me3(Abcam, ab6002), CD44(CST, #3570),CD133, cytokeratin 5 (CK5, Abcam, ab128190), SOX2 (Santa Cruz, sc-8431) and Nanog (CST, #4903), Ki67(Abcam, ab15580).

shRNA and siRNA knockdown studies

The following genes were successfully knocked down using lentiviral vectors purchased from GeneCopoeia (Nts: HSH063249; YAP1: MSH028543; Yap1). Stable knockdown was achieved by selection with 1 mg/ml puromycin. Knockdown (>75%) was confirmed by Western blot analysis. siRNA targeting the YAP1 gene (QIGEN-SI00084567,SI02662954), AR (QIGEN - SI00000679, SI02757258), DNMT3a (QIGEN - SI02665271, SI02665278) and all siRNA negative controls were purchased from QIGEN. Transfections were conducted using Lipofectamine RNAiMAX (Invitrogen).

DNA extraction and modification by sodium bisulfite

Genomic DNAs were isolated from LNCaP cells using the DNeasy tissue kit (Qiagen, Valencia, CA). Bisulfite modification of DNA was performed using the EpiTect ®bisulfite kit (Qiagen, Valencia, CA) following the manufacturer's directions.

Methylation-specific PCR(MSP)

Bisulfite-treated DNAs were amplified by a PCR protocol using the primers described in the Supplementary Table 1. PCR was performed in a volume of 20 µl containing 5× PCR buffer, 1.5 mM MgCl2, 200 M dNTP, 0.32 M of each primer, and 1 unit of Hot Start *Taq* Plus DNA polymerase (Invitrogen). MSP PCR conditions were as follows: 95°C for 300 s, 95°C for 30 s, 52°C for 45 s, 72°C for 45 s for 30 cycles, and 72°C for 180 s. MSP products were purified using the QiaquickTM gel extraction kit (Qiagen, Valencia, CA) and 1:1000 diluted samples were used as MSP template. After the reaction, PCR products were analyzed by gel electrophoresis. As blank controls of MSP reactions, water and unmodified DNAs were used as templates.

In Vitro transcription and T-Cleavage (RNase A Digestion) assay.

Shrimp alkaline phosphatase (SAP; Sequenom) was used to remove unincorporated dinucleotide triphosphates (dNTPs). The components were 1.7 µL of RNase-free ddH$_2$O and 0.3µL of SAP combined with the PCR products. The mixture was incubated at 37°C for 20 min, 85°C for 5 min, and then at 4°C indefinitely. Using T7 R&DNA polymerase (Epicentre, Madison, WI), thymidine triphosphate was incorporated into the PCR product, finishing the transcription reaction. Ribonucleotides and dNTPs were used at concentrations of 1 and 2.5 mM/L, respectively. RNase A (Sequenom) was added to the same reaction to cleave the transcripts (T-cleavage assay). The reaction mixture was incubated at 37°C for 3 h. To remove the phosphate backbone, the T-cleavage/RNase A assay reaction products were diluted with 20µL of RNase-free H$_2$O and mixed with Clean Resin (Sequenom) before performing MS.

Mass spectrometry

A total of 22 nL of the RNase-A treated product was robotically dispensed onto silicon matrix preloaded chips (SpectroCHIP; Sequenom), the mass spectra were collected using a MassARRAY Compact MALDI-TOF (Sequenom), andspectra's methylation ratios were generated by the Epi-TYPER software v1.0 (Sequenom).

MACS

The CD133+/CD44+ PCSLCs were obtained using the MACS kit according to the manufacturer's instructions (Miltenyi Biotech, Bergisch Gladbach, Germany). Briefly, total populations of adherent cells were enzymatically dissociated into a single cell suspension and counted to confirm the quantity of the whole cells. The cells were incubated with 100 μl microbeads directly conjugated to mouse monoclonal anti-human CD133 antibody at 4°C for 30 min. Subsequently, the suspended cells were added to a MACS column that was placed in the magnetic field of a MACS separator (Miltenyi Biotech). The labeled CD133+ cells were retained on the column and the unlabeled cells were eluted; when the column was removed from the magnetic field, the magnetically retained CD133+ cells were collected as positively selected cells for further research. The CD44+ cells were obtained using the same method.

Immunohistochemistry

Tissue sections were de-waxed in xylene and rehydrated in graded alcohol. Antigen retrieval was done under pressure for 5 min in citrate buffer (pH adjusted to 6.0). Endogenous peroxidase was blocked in 0.3% hydrogen peroxide for 10 min and blocked using 1.5% horse serum. Incubation with primary antibody was done in humidified chamber overnight at 4°C (anti-YAP1, 1:100 from Cell Signaling Technology; anti-AR 1:100 from Cell Signaling Technology; Ki67 1:100 from Abcom; SOX2, 1:100 from Santa Cruz and Nanog 1:100 from Cell Signaling Technology). After applying Poly-HRP anti-rabbit IgG (30min), secondary antibody detection was performed using the Ultraview DAB detection kit (Zhongshan Co, China). In the case of YAP1, SOX2, Nanog and Ki67, only nuclear staining was considered as positive and was scored. All immunostained sections were evaluated under Zeiss microscope (×200). At least 10 high power fields around each of the malignant glands were evaluated and scored.

Supplementary Table 1: Data of methylation status among various groups

name:YAP1		HNPC basel	HNPC luminal	CRPC basel	CRPC luminal
**SampleID**	**CPG Position**	**Y1**	**Y2**	**Y3**	**Y4**
YAP1_CpG_1	102	0.47	0.55	0.22	0.3
YAP1_CpG_2	110	0.46	0.65	0.16	0.23
YAP1_CpG_3.4	139:141	0.45	0.57	0.25	0.27
YAP1_CpG_5	153	0.42	0.62	0.26	0.33
YAP1_CpG_6.7.8	161:166:170	0.47	0.41	0.27	0.21
YAP1_CpG_9	179	0.42	0.49	0.32	0.34
YAP1_CpG_10.11	187:189	0.47	0.56	0.19	0.28
YAP1_CpG_12	209	0.48	0.65	0.18	0.3
YAP1_CpG_15	260	0.51	0.55	0.21	0.29
YAP1_CpG_16.17	271:280	0.49	0.52	0.19	0.33
YAP1_CpG_18.19	286:290	0.47	0.62	0.17	0.27
YAP1_CpG_20	304	0.48	0.58	0.18	0.26
YAP1_CpG_21	308	0.46	0.56	0.16	0.32
YAP1_CpG_22	313	0.51	0.62	0.19	0.35
YAP1_CpG_23.24.25.26	338:342:344:351	0.45	0.61	0.25	0.29
YAP1_CpG_27	367	0.53	0.64	0.23	0.32
YAP1_CpG_28	373	0.49	0.63	0.24	0.28
YAP1_CpG_29.30.31.32	387:393:398:340	0.5	0.48	0.2	0.29
YAP1_CpG_33	412	0.58	0.55	0.22	0.27
YAP1_CpG_37.38.39	444:446:450	0.59	0.55	0.23	0.36
YAP1_CpG_40	457	0.59	0.55	0.24	0.32
YAP1_CpG_41	462	0.45	0.63	0.25	0.31
YAP1_CpG_42	472	0.48	0.6	0.26	0.33
YAP1_CpG_43	476	0.46	0.65	0.27	0.34
YAP1_CpG_44.45.46	483:487:491	0.48	0.64	0.18	0.35
YAP1_CpG_47.48.49.50	500:503:505:510	0.52	0.49	0.22	0.28

Supplementary Table 2: Oligonucleotides used in this study

**q-PCR**		
	PSA	Forword primer 5’-ACGCTGGACAGGGGGCAAAAG-3’
		Reverse primer 5’-GGGCAGGGCACATGGTTCACT-3’
	AR	Forword primer 5’-GGAATTCCTGTGCATGAAA-3’
		Reverse primer 5’-CGAAGTTCATCAAAGAATT-3’
	YAP1	Forword primer 5’-GCAACTCCAACCAGCAGCAACA-3’
		Reverse primer 5’-CGCAGCCTCTCCTTCTCCATCTG-3’
	CTGF	Forword primer 5’-GCCACAAGCTGTCCAGTCTAATCG-3’
		Reverseprimer 5’-TGCATTCTCCAGCCATCAAGAGAC-3’
	GAPDH	Forword primer 5’-CCAGCAAGAGCACAAGAGGAAGAG-3’
		Reverse primer 5’-CAAGGGGTCTACATGGCAACTGTG-3’
	SOX2	Forword primer 5’-TTGCTGCCTTAAGACTAGGA-3’
		Reverse primer 5’-CTGGGGCTCAAACTTCTCTC-3’
	OCT4	Forword primer 5’-GACAACAATGAGAACCTTCAGGAGA-3’
		Reverse primer 5’-CTGGCGCCGGTTACAGAACCA-3’
	Nanog	Forword primer 5’-ATCCAGCTTGTCCCCAAAG-3’
		Reverse primer ‘-ATTTCATTCGCTGGTTCTGG-3’
	CD44	Forword primer 5’-TTTGCATTGCAGTCAACAGTC-3’
		Reverse primer 5’-GTTACACCCCAATCTTCATGTCCAC-3’
	CD133	Forword primer 5’-CTGGGGCTGCTGTTTATTA-3’
		Reverse primer 5’-TACCTGGTGATTTGCCACA-3’
	CMET	Forword primer 5’-ACAGTGGCATGTCAACATCGCT-3’
		Reverse primer 5’-GCTCGGTACTCTACAGATTC-3’
	ANKRD	Forword primer 5’-GTGTAGCACCAGATCCATCG-3’
		Reverse primer 5’-CGGTGAGACTGAACCGCTAT-3’
**ChIP-PCR**		
	SOX2	Forword primer 5’-GCGGAGTGGAAACTTTTGTCC-3’
		Reverse primer 5’-CGGGAAGCGTGTACTTATCCTT-3’
	Nanog	Forword primer 5’-CTGACCTCAAGTGATTCACC-3’
		Reverse primer 5’-GCAGAGGAAGCCTTTCAAC-3’
	YAP1	Forword primer 5’-CACATCCTCTCTCCACTTCTTT-3’
		Reverse primer 5’-CTTGGCTGCAGGAAGTTCTT-3’
	PSA	Forword primer 5’-CCTAGATGAAGTCTCCATGAGCTACA-3’
		Reverse primer 5’-GGGAGGGAGAGCTAGCACTTG-3’
MSP		
	M(methylation)	Forword primer 5’-AAGCGTTTTTTTCGAGTACG-3’
		Reverse primer 5’-CGTATTCTACCCCGCGAACC-3’
	UM(unmethylation)	Forword primer 5’-AAGTGTTTTTTTTGAGTATG-3’
		Reverse primer 5’-CATATTCTACCCCACAAACC-3’
Methylation Sequence		
		Forword primer 5’-aggaagagagGTTTATTTTTTTAAGTGAGTTTTTGTAG
		Reverseprimer 3’-cagtaatacgactcactatagggagaaggctCAAATTACTACATTCCTACACACTCC

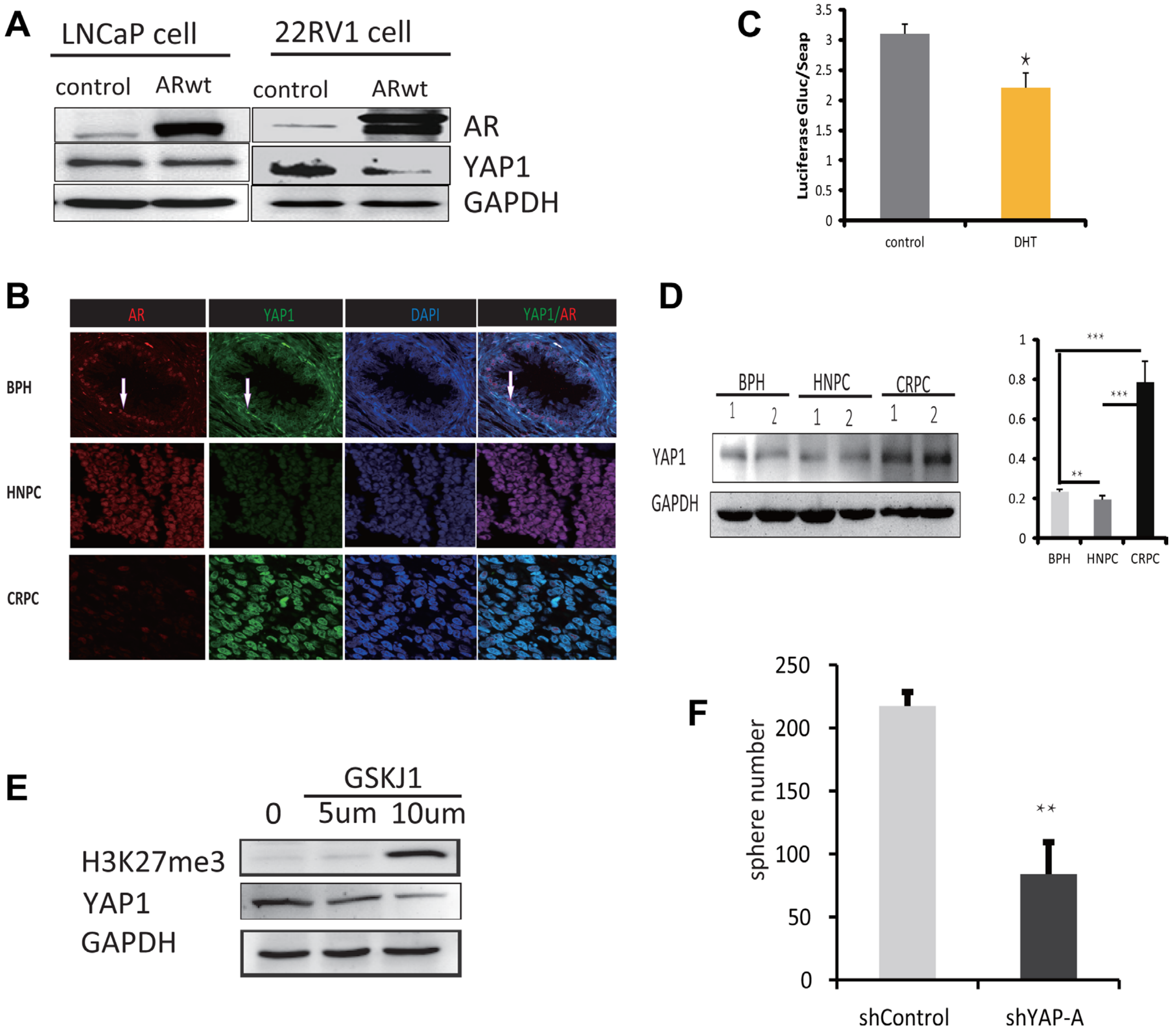

Supplementary Figure 1: AR regulation of YAP1 expression in prostate cancer cells (related to Figure 1). (**A**) YAP1 protein levels were analyzed by Western Blot (WB) in LNCAP and 22rv1 cells transfected with an AR expression vector as indicated (**B**) Immunofluorescence staining (IF) and quantitation of AR (red) and YAP1 (green) in BHP, HNPC and CRPC tissue sourced from human prostate cancer patients. Cell nuclei were visualized by DAPI staining. (**C**) Yap1 gene promoter driven Luciferase reporter activity measured after DHT treatment in LNCaP cell. Statistical significance was analyzed by Student's T-Test, ($^{*}p < 0.05$). (**D**) YAP1 protein expression analyzed by Western blotting on BPH and HNPC and CRPC samples obtained from different patients. (**E**) WB analysis of YAP1 and H3K27me3 protein expression in LNCaP cells treated with GSKJ1. (**F**) Sphere formation assay in C4-2 cells transfected with sh-control and shYAP expressing vectors. Quantitation of the numbers of spheres formed are shown as the mean +/-SD. *P* values were determined by paired *T*-test. $^{*}p < 0.05$, $^{**}p < 0.01$, $^{***}p < 0.001$.

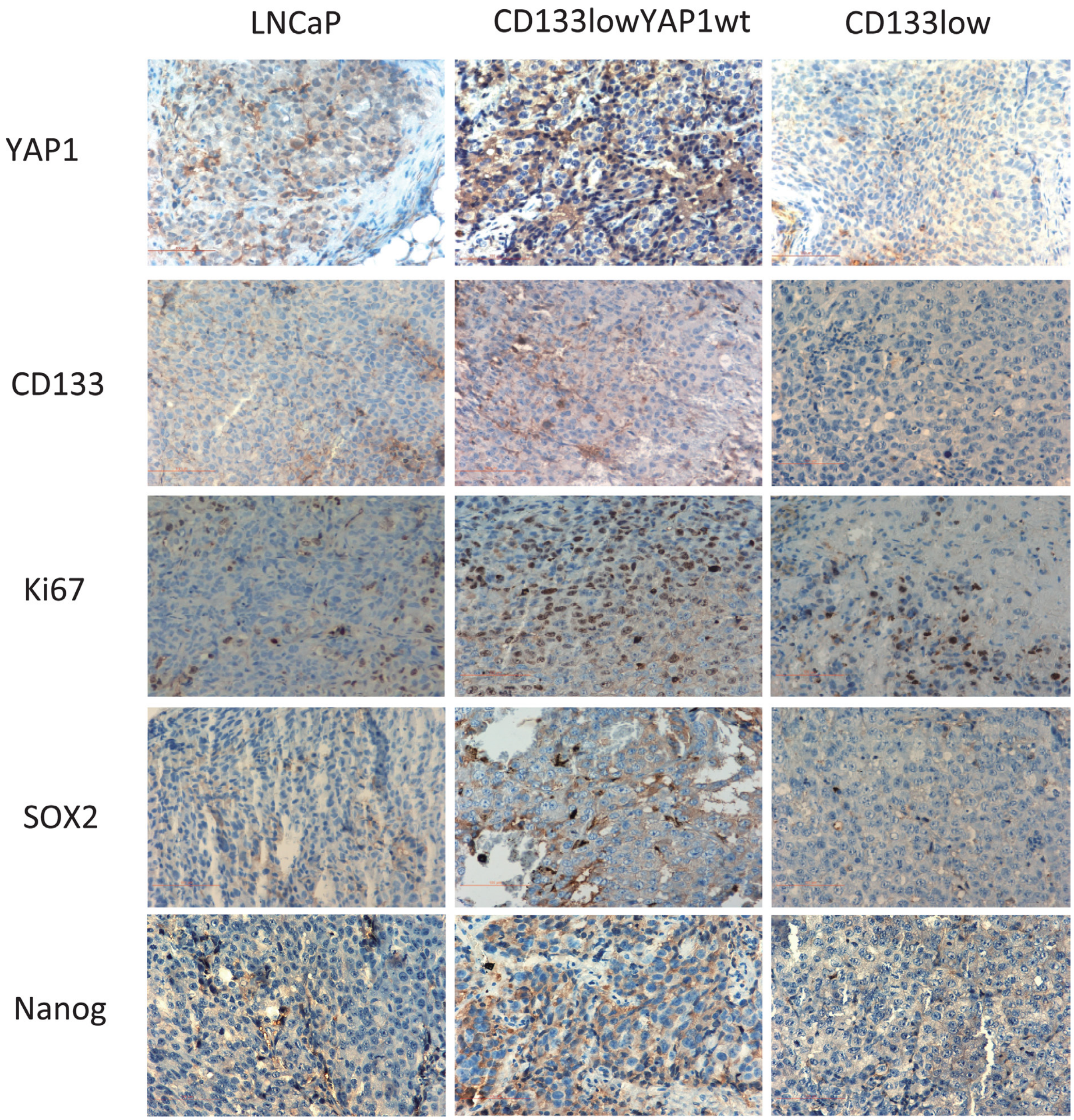

Supplementary Figure 2: YAP1 promote PC cell self renewal *in vivo,* related to Figure 4. Unselected LNCaP cells, LNCAP-CD133^{low} cells and LNCAP-CD133^{low} stably transfected with a YAP1 expression vector (CD133^{low}-YAP1wt) cells were transplanted subcutaneously into nude mice. IHC was used to visualize the expression of YAP1, Ki67, CD133, SOX2 and Nanog proteins in tumor tissue.

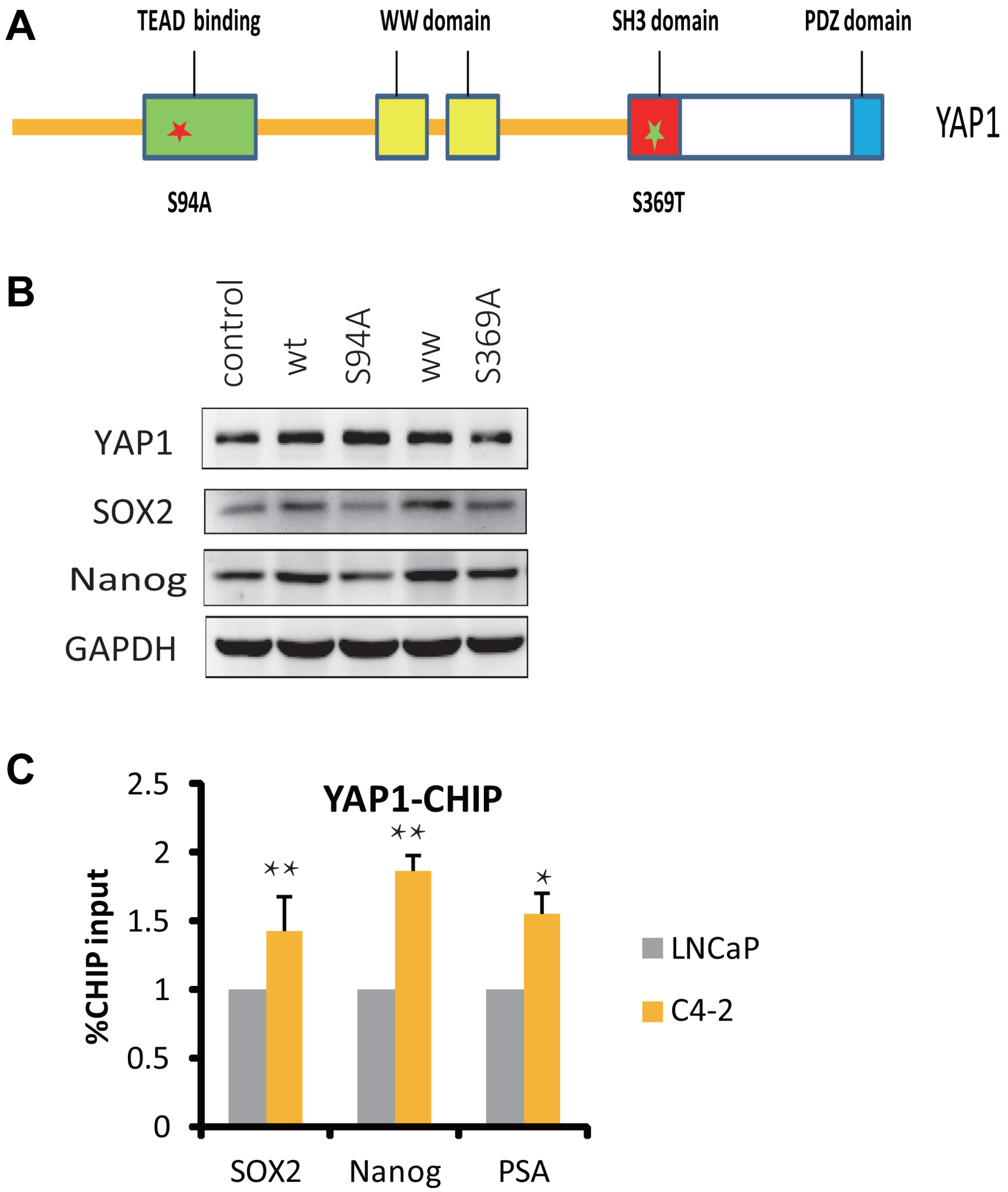

Supplementary Figure 3: Structure-function analysis of YAP1 function, related to Figure 5. (**A**) Cartoon representation of the YAP1 domain structure and YAP1 mutants. (**B**) Western blot analysis of SOX2 and Nanog protein expression in C4-2 cell stably transduced with expression vectors for YAP1-WT, and YAP1 S94A, WW domain, and S369A mutants. (**C**) ChIP–qPCR analysis YAP1 binding to the SOX2, Nanog and PSA genes promoter.

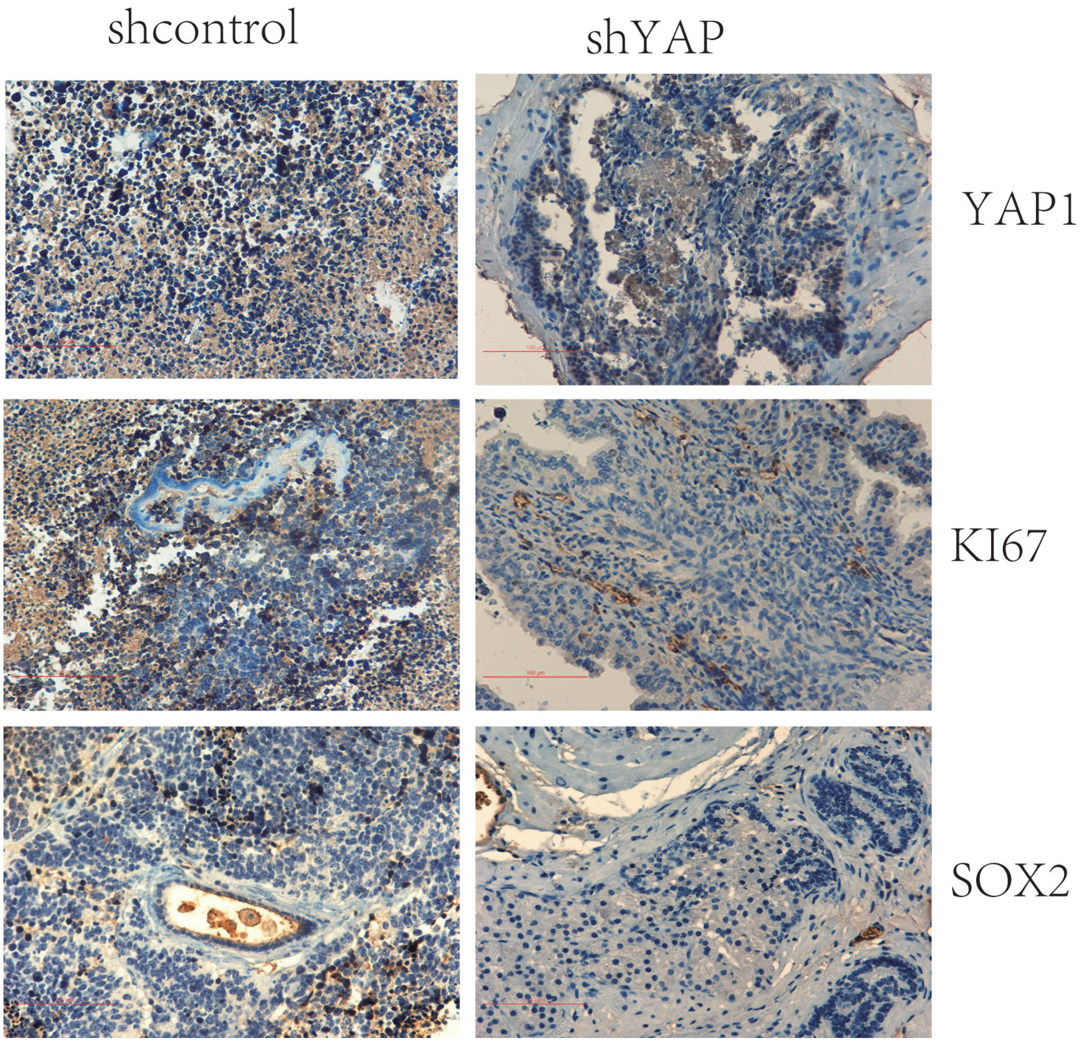

Supplementary Figure 4: YAP1, Ki67and SOX2 protein expression in TRAMP mice after lentivirus-shYAP1 treatment, related to Figure 6. Representative images of YAP1, Ki67 and SOX2 protein expression in TRAMP mice tumors after lentivirus-shYAP1 therapyS specimens were obtained 2 weeks post infection.

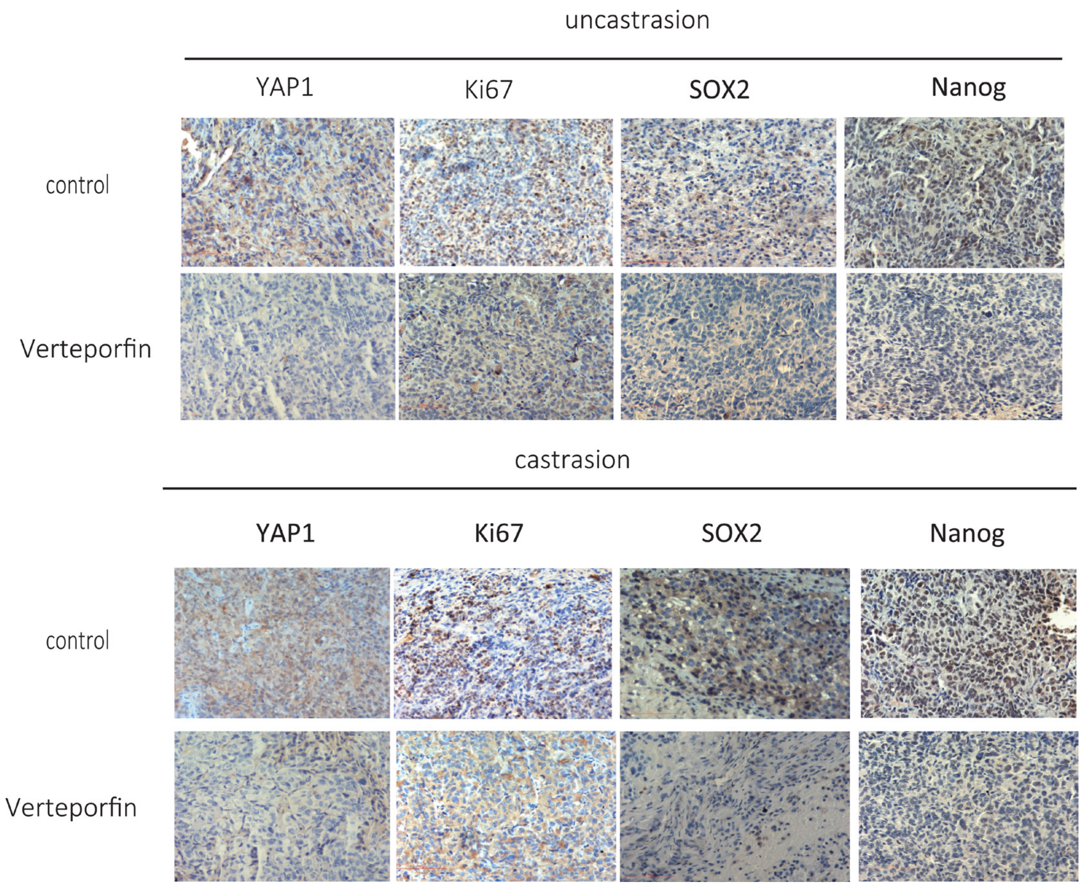

Supplementary Figure 5: YAP1, Ki67, SOX2 and nanog protein expression in LNCaP cell xenograft, related to Figure 6. Representative images of YAP1, Ki67, SOX2 and Nanog protein expression in LNCaP xenograft tumors grown in either naïve treated or castrated mice after Verteporfin therapy. Specimens were collected 10 days post-treatment.

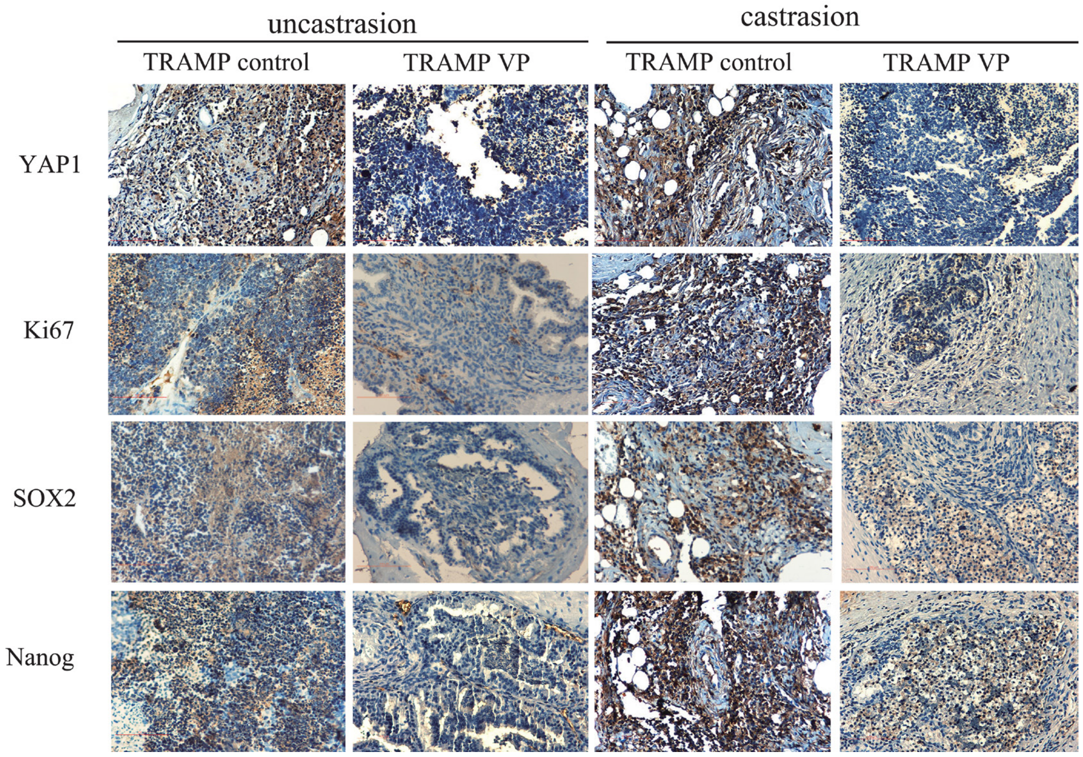

Supplementary Figure 6: YAP1, Ki67, SOX2 and nanog protein expression in TRAMP mice, related to Figure 6. Representative images of YAP1, Ki67, SOX2 and Nanog protein expression in LNCaP xenograft tumors grown in either naïve treated or castrated mice after Verteporfin therapy. Specimens were obtained 2 weeks post-treatment.
